# A heterogeneous graph convolutional attention network method for classification of autism spectrum disorder

**DOI:** 10.1186/s12859-023-05495-7

**Published:** 2023-09-27

**Authors:** Lizhen Shao, Cong Fu, Xunying Chen

**Affiliations:** 1 Beijing Engineering Research Center of Industrial Spectrum Imaging, School of Automation and Electrical Engineering, Beijing, 100083 China; 2 Lancaster University, Lancaster, LA1 4YX UK; 3 Shunde Graduate School, University of Science and Technology Beijing, Foshan, 528399, China

**Keywords:** FMRI, Heterogeneous graph convolution network, ASD, Attention mechanism

## Abstract

**Background:**

Autism spectrum disorder (ASD) is a serious developmental disorder of the brain. Recently, various deep learning methods based on functional magnetic resonance imaging (fMRI) data have been developed for the classification of ASD. Among them, graph neural networks, which generalize deep neural network models to graph structured data, have shown great advantages. However, in graph neural methods, because the graphs constructed are homogeneous, the phenotype information of the subjects cannot be fully utilized. This affects the improvement of the classification performance.

**Methods:**

To fully utilize the phenotype information, this paper proposes a heterogeneous graph convolutional attention network (HCAN) model to classify ASD. By combining an attention mechanism and a heterogeneous graph convolutional network, important aggregated features can be extracted in the HCAN. The model consists of a multilayer HCAN feature extractor and a multilayer perceptron (MLP) classifier. First, a heterogeneous population graph was constructed based on the fMRI and phenotypic data. Then, a multilayer HCAN is used to mine graph-based features from the heterogeneous graph. Finally, the extracted features are fed into an MLP for the final classification.

**Results:**

The proposed method is assessed on the autism brain imaging data exchange (ABIDE) repository. In total, 871 subjects in the ABIDE I dataset are used for the classification task. The best classification accuracy of 82.9% is achieved. Compared to the other methods using exactly the same subjects in the literature, the proposed method achieves superior performance to the best reported result.

**Conclusions:**

The proposed method can effectively integrate heterogeneous graph convolutional networks with a semantic attention mechanism so that the phenotype features of the subjects can be fully utilized. Moreover, it shows great potential in the diagnosis of brain functional disorders with fMRI data.

## Backgound

Autism spectrum disorder (ASD) is a developmental disability that can cause significant social, communication and behavioral challenges [1]. ASD has attracted great attention from neuroscientists and clinical scientists, who hope to clarify its pathogenic mechanism and find an effective treatment method [2]. For children with ASD, early identification and intervention are important since they may mitigate disease severity and ameliorate the quality of the patients’ lives. However, due to the complexity and heterogeneity of ASD, no effective biomarkers for ASD have been found at present. The diagnosis of ASD is mainly based on the interaction between individuals and clinicians [3, 4]. Many children cannot receive a final diagnosis until much older.

In the past decade, functional magnetic resonance imaging (fMRI) as a promising neuroimaging technique has been widely used for studying interregional functional connectivity (FC) in the human brain. In fMRI studies, FC is defined as the temporal correlation of blood oxygen level dependent signals measured in various brain regions. It is used to identify potential neuroimaging biomarkers for the diagnosis of neurological diseases [5, 6]. In some specific functional connectivity in the brains with ASD, abnormalities have been found. For instance, Monk et al. [7] discovered that intrinsic connectivity within the default network in ASD subjects has been altered, and that connectivity between these structures is related to specific ASD symptoms. Therefore, effective modelling with brain functional connectivity of fMRI data is conducive to the identification of biomarkers for ASD.

Based on fMRI data, many machine learning methods and deep learning methods have been proposed for ASD classification. Feng et al. [8] summarized the progress of ASD classification work with the Autism Brain Imaging Data Exchange (ABIDE) dataset in the last three years. Kong et al. [9] proposed an ASD-assisted diagnosis method based on a deep neural network (DNN). Mostafa et al. [10] proposed diagnosing ASD based on eigenvalues of brain networks and linear discriminant analysis (LDA). Ahmed et al. [11] designed a single volume image generator that converts individual fMRI images into a series of 2-dimensional images. Then they used an improved convolutional neural network to classify those generated images. Guo et al. [12] proposed a sparse autoencoder based feature selection method, and developed a DNN-based classification model for distinguishing ASD patients from typically developed controls. Heinsfeld et al. [13] extracted low-dimensional features from training samples with two stacked denoising autoencoders. Then they used an MLP to classify ASD and achieved a classification accuracy of 70% on the ABIDE dataset. Eslami et al. [14] proposed a framework called ASD-DiagNet to classify ASD by using only fMRI data. Hu et al. [15] proposed an interpretable fully connected neural network (FCNN) to identify ASD participants from fMRI data and obtained an accuracy of 69.81%. Liu et al. [16] improved ASD classification using dynamic functional connectivity (DFC) and multitask feature selection. They used a multikernel support vector machine (SVM) learning method for ASD classification and achieved an accuracy of 76.8% on the ABIDE I dataset. Brahim and Farrugia [17] presented an approach based on graph fourier transform (GFT) and SVM for the analysis of resting-state functional magnetic resonance imaging. Yin et al. [18] employed an autoencoder (AE) to learn advanced features from fMRI data. Then they trained a DNN with the learned features and achieved a classification accuracy of 76.2%. Haghighat et al. [19] proposed an age-dependent connectivity-based ASD computer aided diagnosis system using resting state fMRI. Wang et al. [20] proposed a multisite clustering and nested feature extraction (MC-NFE) method for fMRI-based ASD detection. Experimental results on 609 subjects from the ABIDE database suggest that the proposed MC-NFE outperforms several state-of-the-art methods in ASD detection.

Recently, graph neural networks, which generalize deep neural network models to graph structured data, have shown great advantages in model training and classification tasks [21]. Researchers have tried to classify ASD data using graph models. In 2017, Parisot et al. [22] constructed a population graph using fMRI and phenotypic data, in which nodes and arc weights are associated with image-based feature vectors and phenotypic data, respectively. Then they applied a graph convolutional network (GCN) with the population graph as input to classify ASD. The results showed that integrating phenotypic data in classification tasks was beneficial. In 2018, Parisot et al. [23] further studied the impact of different feature selection strategies on the classification of ASD. They used a GCN in a semisupervised manner for node classification. A classification accuracy of 70.4% for the ABIDE dataset was achieved. Rakhimberdina et al. [24] proposed a population graph-based multimodel ensemble to classify patients with ASD and healthy controls (HCs). Compared with using a single model, the proposed method obtained higher accuracy on the ABIDE dataset. Jiang et al. [25] proposed a hierarchical GCN framework to learn graph feature embeddings for ASD classification. In the framework, the network topology information and subject’s association are considered at the same time. Li et al. [26] proposed a graph neural network framework (BrainGNN) to analyse functional magnetic resonance images and discovered neurological biomarkers for ASD. Wen et al. [27] presented a prior brain structure learning-guided multiview graph convolutional neural network to learn common features for ASD classification. In our previous work [28], a combination of deep feature selection and GCN was proposed to classify ASD. First, the deep feature selection method of [29] was used to select the functional connection features of fMRI data. Then, a GCN was used to classify 871 subjects in the ABIDE I dataset, and a high classification accuracy of 79.5% was achieved, which is currently the highest.

As brain connectivity graphs are irregular graph structures, GCNs are well suited to handle such data structures. Thus, the classification performances of the above methods are significantly improved compared to traditional machine learning methods. However, it needs to be noted that in the above graph-based models for ASD classification, the graphs constructed are all homogeneous (i.e., only one type of node and one type of arc are constructed) in which the imaging features are mapped into node feature vectors while the phenotype features are mapped into arc weights. However, since arc weights are scalar, they cannot fully represent the phenotype features. Therefore, the performances still suffer from the limitation that all edges in the graph have an aggregated weight and the phenotypic data are not fully used. To solve this problem, this paper further investigates using graph neural networks to classify ASD patients from healthy controls. The goal of the present work is to fuse fMRI and phenotype information of subjects into a graph neural network so that better classification performance and more accurate diagnosis can be achieved.

In order to fully make use of the phenotype information of non-imaging data of the subjects, a heterogeneous population graph based on the fMRI and phenotypic data is constructed. At the same time, an attention mechanism is introduced so that different weights can be learned and aggregated important features can be extracted. Therefore, based on the heterogeneous graph, GCN and attention mechanism, a heterogeneous graph convolution attention network (HCAN) for the classification of ASD is proposed. This work is inspired by the work of [30], a heterogeneous graph attention network for node classification. Different from homogeneous graphs, heterogeneous graphs have multiple types of nodes and arcs. In HCAN, different phenotype features are mapped into different types of arcs; thus, richer hidden information is contained.

The main contribution of this work is summarized as follows.

- In this paper, a heterogeneous graph construction method is constructed for the ABIDE dataset. The heterogeneous graph contains not only imaging data features but also rich phenotypic data features.
- Based on the heterogeneous graph, a heterogeneous graph convolution attention network for ASD classification is proposed. With the attention mechanism, the importance of phenotype information can be fully considered.
- On the ABIDE dataset, the proposed method achieves the best classification accuracy of 82.9%, which is the new state-of-the-art and significantly outperforms previous approaches.

The rest of the paper is organized as follows. In Sect. 2, the ABIDE dataset and the preprocessing of the data are introduced. In Sect. 3, the proposed HCAN method, including the construction of a heterogeneous graph, the heterogeneous graph convolution network, the semantic attention network, and the model loss function, is shown. In Sect. 4, some numerical results are shown, and the proposed method is compared with some other methods in the literature. Finally, conclusions are drawn in Sect. 5.

## Data and preprocessing

This paper carries out research on the challenging public ABIDE I dataset [31], which aggregates data from 17 different international collection sites, sharing neuroimaging and phenotype data of 1112 subjects. In the experiment, 871 subjects (including 403 ASD patients and 468 healthy controls) who meet the imaging quality and atypical information criteria were used. The related phenotypic data, including ‘Age’, ‘Handedness’, and ‘Sex’ of these subjects are shown in Table 1.

**Table 1:** Phenotype data of the selected 871 subjects in the ABIDE I dataset for individual site

Site	ASD			TD		
	Age	Handedness (L/R/Other)*	Sex (M/F)	Age	Handedness (L/R/Other)*	Sex (M/F)
CALTECH	24.0 ± 7.6	0/4/1	4/1	28.2 ± 12.2	1/9/0	6/4
CMU	26.0 ± 5.4	1/5/0	4/2	27.8 ± 4.4	0/5/0	3/2
KKI	10.7 ± 1.3	0/9/3	9/3	10.1 ± 1.2	1/18/2	15/6
LEUVEN_1	21.9 ± 4.1	1/13/0	14/0	23.0 ± 2.8	1/13/0	14/0
LEUVEN_2	13.9 ± 1.5	2/10/0	9/3	14.4 ± 1.5	3/13/0	12/4
MAX_MUN	28.4 ± 13.2	2/17/0	16/3	25.2 ± 8.4	0/27/0	26/1
NYU	14.8 ± 7.1	0/0/74	64/10	15.8 ± 6.2	0/0/98	72/26
OHSU	11.4 ± 2.2	1/11/0	12/0	10.2 ± 1.0	0/13/0	13/0
OLIN	17.1 ± 3.3	3/11/0	11/3	16.9 ± 3.6	2/12/0	12/2
PITT	18.3 ± 7.0	3/21/0	21/3	18.7 ± 6.7	1/24/1	22/4
SBL	34.0 ± 6.6	1/0/11	12/0	33.6 ± 6.8	0/0/14	14/0
SDSU	15.3 ± 1.8	0/8/0	8/0	14.0 ± 1.9	2/17/0	13/6
STANFORD	10.2 ± 1.6	3/8/1	9/3	9.8 ± 1.7	0/12/1	9/4
TRINITY	17.0 ± 3.2	0/19/0	19/0	17.1 ± 3.8	0/25/0	25/0
UCLA_1	13.3 ± 2.6	3/34/0	31/6	13.4 ± 2.1	3/24/0	24/3
UCLA_2	12.8 ± 2.0	3/8/0	11/0	12.1 ± 1.2	0/10/0	8/2
UM_1	13.3 ± 2.5	5/25/4	26/8	14.1 ± 3.2	7/42/3	35/17
UM_2	14.9 ± 1.6	1/11/1	12/1	16.7 ± 4.0	2/19/0	20/1
USM	23.6 ± 8.4	0/0/43	43/0	20.9 ± 8.3	0/0/24	24/0
YALE	13.1 ± 3.0	5/17/0	14/8	13.6 ± 2.1	2/17/0	11/8
Total	17.1 ± 8.0	34/231/138	349/54	16.8 ± 7.2	25/300/143	378/90

* Handedness: ‘L’ represents ‘Left’, ‘R’ represents ‘Right’, ‘Other’ includes ‘Mixed’, ‘Ambidexterous’ and ‘Not avaliable’

The preprocessed data of the 871 subjects were downloaded from the Preprocessed Connectomes Project (http://preprocessed-connectomes-project.org/). Data preprocessing was performed using the configurable pipeline for the analysis of connectomes. According to the Harvard-Oxford atlas, there are 111 ROIs in the brain [32]. The mean time series for each ROI was calculated. Then the distance correlation coefficients between different mean time series were calculated to obtain a functional connection matrix. Finally, the 6105 elements belonging to the upper right triangle part of the matrix were extracted to form a functional connection feature vector.

## The proposed method

**Fig. 1:**
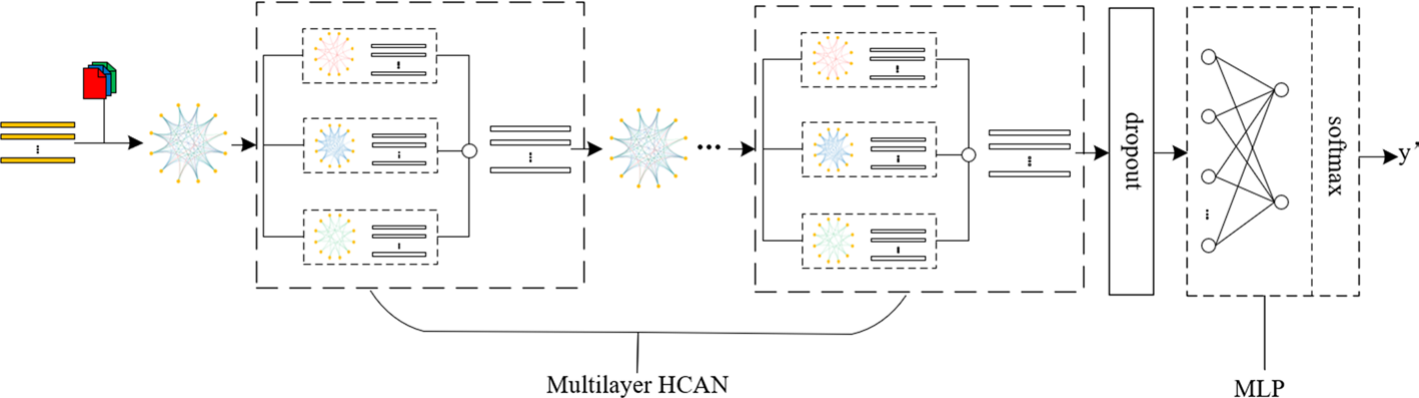
The architecture of the HCAN model, which inludes a multilayer HCAN and an MLP

In this section, the proposed HCAN method for the classification of ASD is introduced. The architecture of the proposed HCAN model is shown in Fig. 1, which includes a multilayer HCAN and an MLP. The input of the model is fMRI and phenotypic data, while the output is the prediction result (i.e., the probability of ASD) of each sample.

For a specified classification task, the HCAN model works as follows. First, a heterogeneous population graph using the fMRI and phenotypic data is constructed. Then, the heterogeneous graph is processed through a multilayer HCAN to extract fused features with semantic information. Next, the fused features will go through a dropout layer for regulation and are further fed into an MLP with softmax to output prediction results.

**Fig. 2:**
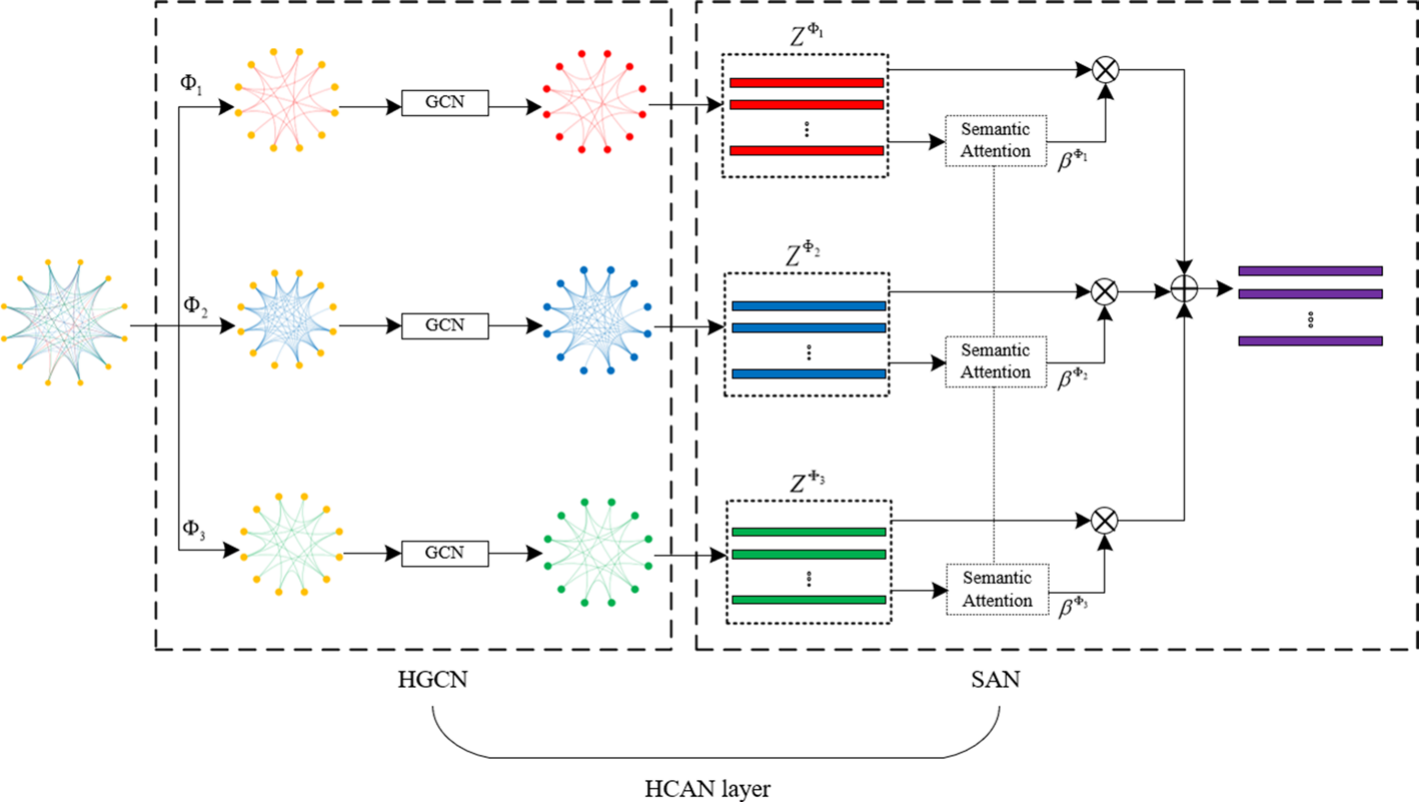
The structure of a HCAN layer. Each HCAN layer consists of a heterogeneous graph convolutional network (HGCN) and a semantic attention network

The structure of an HCAN layer is shown in Fig. 2. Each HCAN layer consists of a heterogeneous graph convolutional network (HGCN) and a semantic attention network (SAN).

Next, the proposed method will be shown in detail from the following three parts: the construction of a population heterogeneous graph, the HCAN model, and the loss function of the model.

### Heterogeneous graph construction

Different from homogeneous graphs, heterogeneous graphs are a special type of information network that involve multiple types of objective nodes or multiple types of arcs [33].

#### Definition 1

([33]) Heterogeneous graph $G = (V, E)$ consists of a node set *V* and an arc set *E*. Moreover, there is a mapping relationship $\phi :V \rightarrow Q$, and $\psi :E \rightarrow S$, where *Q* is the node type collection, *S* is the arc type collection, and $|Q|+|S|>2$.

For a heterogeneous graph, two objective nodes can be connected through different semantic paths. These paths are called meta-paths.

#### Definition 2

([34]) For a heterogeneous graph *G*, a meta-path $\Phi$ is defined as: $Q_1\xrightarrow {S_1} Q_2\xrightarrow {S_2} \dots \xrightarrow {S_l} Q_{l+1}$ ($Q_1Q_2\dots Q_{l+1}$). It represents a composite relation $S=S_1\circ S_2 \circ \dots \circ S_l$ between node $Q_1$ and node $Q_{l +1}$, and $\circ$ refers to composition operator on relations.

In a heterogeneous graph, the relations defined by different meta-paths are different, and they can be used to analyse the composite connections and meanings between different nodes. Given a meta-path, for each node, its neighbor nodes are defined as all the other nodes on the path. A set of neighbors based on the meta-path contains structure information and specific semantics.

This paper constructs a heterogeneous population graph of the ABIDE dataset, where image-based functional connection features are contained in the nodes, while non-image phenotype features are contained in the arcs. In the graph, there is only one type of node (i.e., sample nodes) being constructed. There is a one-to-one corresponding relationship between the nodes and the samples. Each node contains an image-based feature vector of a sample. For each sample, the functional connection feature vector after feature selection can be used as the feature vector of the sample node.

**Fig. 3:**
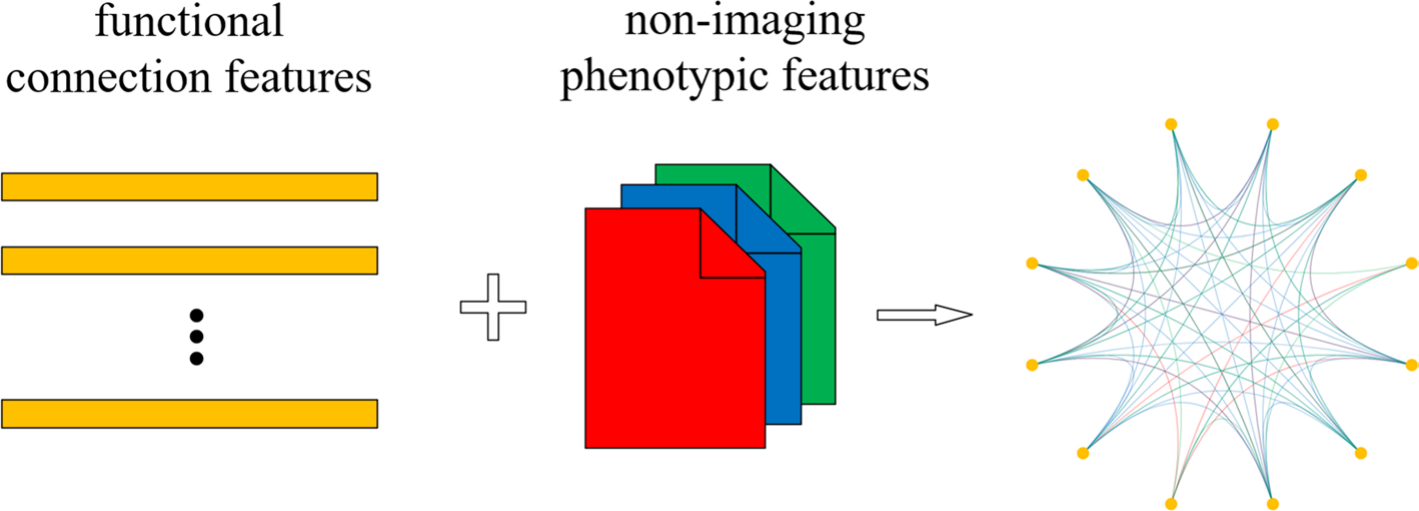
Construction of a heterogeneous graph with functional connection features and non-image phenotype features. Image-based functional connection features are contained in the nodes, while non-image phenotype features are contained in the arcs

Once the sample nodes are set, they are connected by different arcs according to the non-image phenotype features of the samples. Specifically, according to a certain type of non-image phenotype feature, the samples with the same non-image phenotype attribute value are connected. Therefore, the number of arc types is equal to the number of involved non-image phenotype features. In this work, three types of arcs based on ‘site’, ‘sex’, and ‘handedness’ are constructed. For example, if a non-image phenotype feature is ‘sex’, all the samples with the sex of ‘male’ are connected, while all the samples with the sex of ‘female’ are connected, and those connections are regarded as the arcs of the ‘sex’ type. All the arcs are undirected and unweighted, which forms an undirected unweighted heterogeneous graph. Figure 3 shows the construction of a heterogeneous population graph based on the ABIDE dataset, in which red, blue, and green are used to distinguish the three types of arcs based on ‘site’, ‘sex’, and ‘handedness’, respectively.

### Heterogeneous graph convolutional networks

Graph convolutional networks are important tools for graph data feature extraction. However, graph convolutional networks can only be used for training homogeneous graphs. Therefore, this research designs a heterogeneous graph convolutional network (HGCN) to extract features from heterogeneous graphs. The HGCN includes the decomposition of a heterogeneous graph and residual graph convolution networks.

In an HGCN, the constructed heterogeneous graph is first decomposed into several homogeneous graphs based on the meta-paths. Then, for each homogeneous graph, an independent residual graph convolution network is set up. Thus, for each sample node in the heterogeneous graph, different embedding vectors (representations) can be obtained through the forward propagation of different residual graph convolution networks, and they can be integrated as a weighted sum fused feature vector.

#### Decomposition of a heterogeneous graph

In a heterogeneous graph, sample nodes are connected with different types of arcs based on meta-paths. The neighbor connections represent a certain type of relation between the samples. The connected nodes have more potential similar features than the unconnected ones. For example, if two sample nodes are connected based on the ‘node - sex - node’ meta-path, then the two samples have the same ‘sex’ attribute. To fully use and mine the structure information and specific semantics information in a meta-path, the heterogeneous graph is decomposed into multiple homogeneous graphs based on meta-paths.

**Fig. 4:**
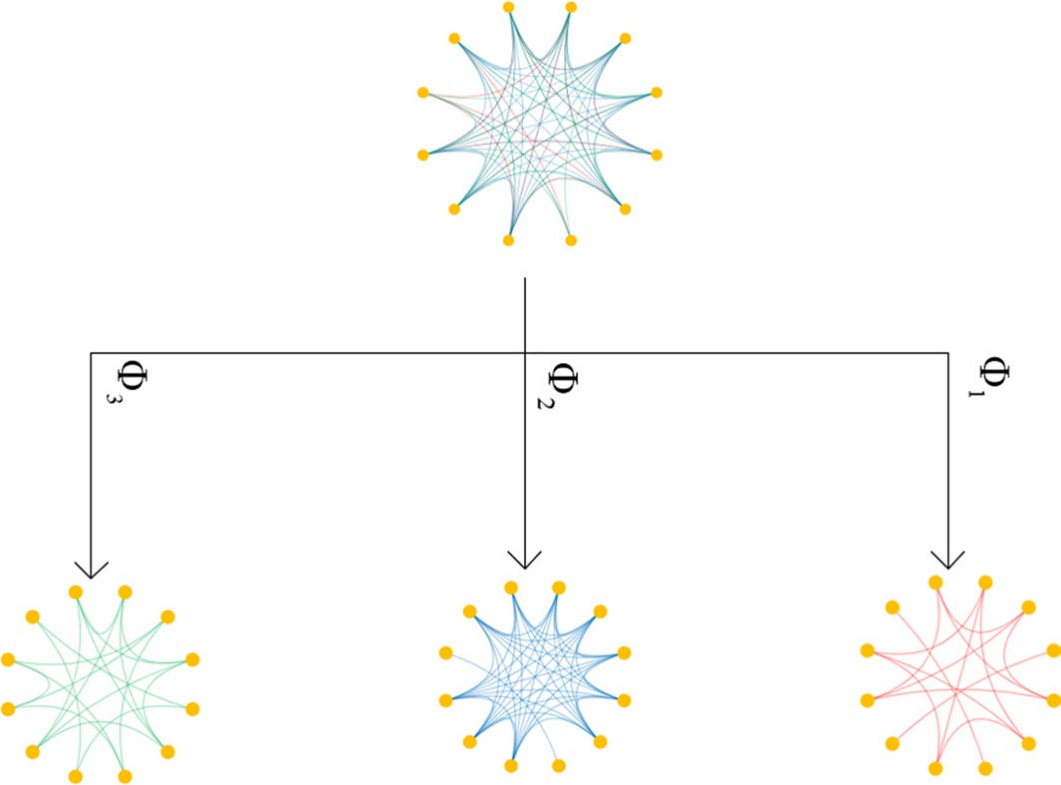
Decomposition of a heterogeneous graph into homogeneous graphs based on meta-paths

For a specific meta-path, when a node is connected with all its neighbor nodes in a new graph, a homogeneous graph can be obtained. For the ABIDE heterogeneous population graph, based on the three types of meta-paths, i.e., ‘node - sex - node’, ‘node - site - node’, and ‘node - handedness - node’, three homogeneous graphs (see Fig. 4) can be obtained. It needs to be noted that all the nodes with their feature vectors in the homogeneous graph are inherited from the heterogeneous graph.

#### Residual graph convolutional networks

For each homogeneous graph, a residual graph convolutional network is constructed to extract features. Consider an undirected unweighted graph $G = (V, E, A)$, where *V* is a node set, $\left| V \right| =n$, *E* is an arc set, and $A \in {\mathbb {R}}^{N\times N}$ is the adjacency matrix. Let *D* be the degree matrix and *L* be the normalized graph Laplacian; then, $L=I_N - D^{-\frac{1}{2}}AD^{-\frac{1}{2}}$, where $I_N \in {\mathbb {R}}^{N\times N}$ is an identity matrix. *L* can be decomposed as $L=U\Lambda U$ with the matrix of eigenvectors *U* and the diagonal matrix of its eigenvalues $\Lambda$. Suppose that each node *i* in the graph contains only one-dimensional feature $x_i$, then the vector signal formed for all the nodes is $x\in {\mathbb {R}}^N$. Let us consider spectral convolutions on graphs defined as the multiplication of signal *x* with a filter (convolution kernel function) $g_{\theta } = diag(\theta )$ parameterized by $\theta \in {\mathbb {R}}^N$ in the Fourier domain

$$\begin{aligned} g_{\theta }*x=Ug_{\theta }U^Tx. \end{aligned}$$

In view of the high computational complexity of graph convolution operations, the Chebyshev polynomial expansion method can be applied to approximate the convolution kernel function $g_{\theta }$. Usually, a first-order Chebyshev approximation is adopted. Thus, the convolution operation of a graph signal can be approximated as follows:

$$\begin{aligned} g_{\theta }*x\approx \theta '{{\tilde{D}}}^{-\frac{1}{2}}{\tilde{A}}{\tilde{D}}^{-\frac{1}{2}}x, \end{aligned}$$

where $\theta '$ is a convolution kernel parameter, $\tilde{A}=A+I_N$, ${{\tilde{D}}}$ is a diagonal matrix, and $\tilde{D}_{ii}=\sum _j{\tilde{A}}_{ij}$. At this point, the graph convolution expression of the one-dimensional signal on the graph is obtained. Since each node may contain multiple features, i.e., the signal on a node is multi-channel, the one-dimensional signal *x* is generalized to be *C* channel signals $X \in {\mathbb {R}}^{N\times C}$. Suppose there are *F* convolution kernels (the number of convolution kernels is also denoted as the hidden size of an HCAN layer), the convolution operation for *X* is as follows:

$$\begin{aligned} Z={{\tilde{D}}}^{-\frac{1}{2}}{\tilde{A}}{{\tilde{D}}}^{-\frac{1}{2}}X\Theta , \end{aligned}$$

where $\Theta$ is a matrix of convolution kernel parameters, and $Z \in {\mathbb {R}}^{N\times F}$ is the convolved signal matrix.

Therefore, the graph convolutional network has the following layer-wise propagation rule,

$$\begin{aligned} H^{(l+1)}=\sigma ({{\tilde{D}}}^{-\frac{1}{2}}{\tilde{A}}{\tilde{D}}^{-\frac{1}{2}}H^{(l)}W^{(l)}), \end{aligned}$$

where $H^{(l)}\in {\mathbb {R}}^{N\times D}$ is the output of the *l*th layer of the network ($H^{(0)}=X$), $\sigma$ denotes an activation function such as $ReLU(\cdot )=max(0,\cdot )$, and $W^{(l)}$ is the network parameter of the *l*th layer, which can be trained. Considering that the graph convolutional network is difficult to train, a residual connection is added to the graph convolutional network; thus, the above layer-wise propagation rule is changed to

$$\begin{aligned} H^{(l+1)}=\sigma ({{\tilde{D}}}^{-\frac{1}{2}}\tilde{A}{\tilde{D}}^{-\frac{1}{2}}H^{(l)}W^{(l)}) + H^{(l)}M, \end{aligned}$$

where *M* is a linear transformation matrix. When the dimensions of $H ^ {(l)}$ and $H ^ {(l+1)}$ are the same, *M* is an identity matrix.

### Semantic attention networks

**Fig. 5:**
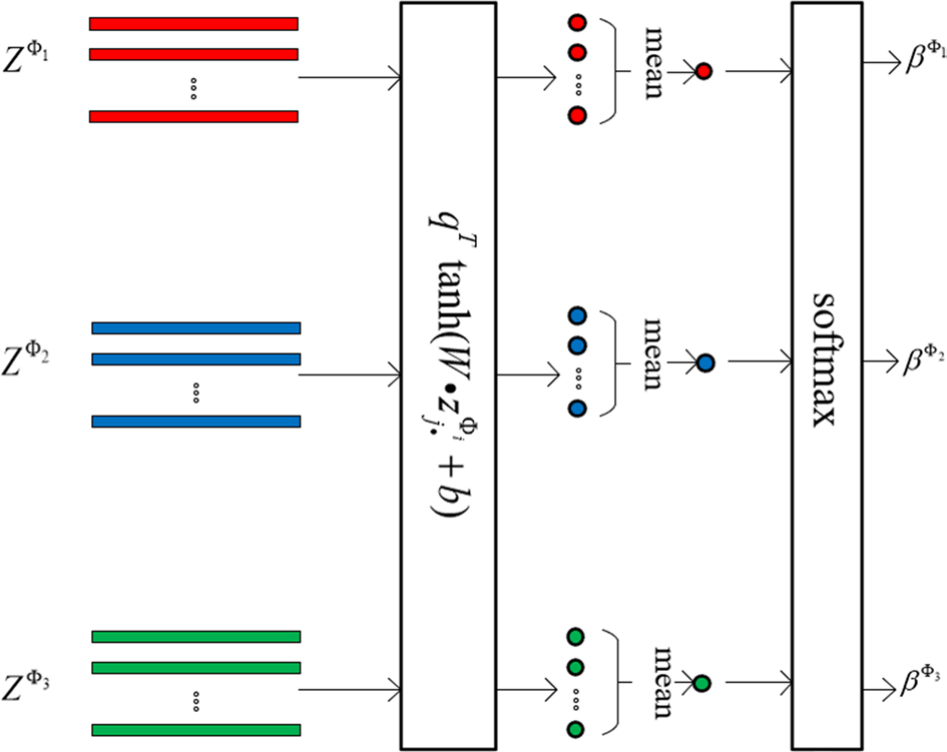
Computation of attention weight $\beta ^{\Phi _i}$ for embedding vector $Z^{\Phi _i}$ in a semantic attention network

For each sample node, after forward propagation through the heterogeneous graph convolutional network, three embedding vectors can be obtained. Each embedding vector contains a piece of specific semantic information, which is related to its corresponding meta-path. Since the importance of that semantic information to the classification task is difficult to determine, a semantic-level attention network is constructed to learn the importance of different semantic information. Based on the three meta-paths, the attention weights for the three specific semantics are

$$\begin{aligned} (\beta ^{\Phi _1},\beta ^{\Phi _2},\beta ^{\Phi _3})=attsem(Z^{\Phi _1},Z^{\Phi _2},Z^{\Phi _3}), \end{aligned}$$

where $Z^{\Phi _1},Z^{\Phi _2}$ and $Z^{\Phi _3}$ represent the embedding vectors of all the sample nodes obtained based on meta-paths $\Phi _1$, $\Phi _2$, and $\Phi _3$, respectively, and $attsem (\cdot )$ represents the neural network for computing attention weights (which can be used to learn the importance of each semantic information through back-propagation). The process of computing semantic attention weights is shown in Fig. 5.

Let $z_{j\cdot }^{\Phi _i}$ be the *j*th row of $Z^{\Phi _i}$, an embedded vector of node *j* $(j \in V)$ based on meta-path $\Phi _i$. It contains specific semantic information related to meta-path $\Phi _i$. In a semantic attention network, first, the embedding vector $z_{j\cdot }^{\Phi _i}$ is transformed into an embedding representation of the specific semantic through a learnable nonlinear transformation

$$\begin{aligned} tanh(Wz_{j\cdot }^{\Phi _i}+b), \end{aligned}$$

where *W* is a weight matrix, and *b* is an offset vector. Then, a learnable semantic-level attention vector *q* is used to measure the importance of the specific semantic by calculating the similarity between the embedding representation $tanh(Wz_{j\cdot }^{\Phi _i}+b)$ and the semantic-level attention vector *q*. Next, for the specific semantic based on meta-path $\Phi _i$, the average of those importance factors of all the nodes $w^{\Phi _i}$ is calculated with

$$\begin{aligned} w^{\Phi _i} = \frac{1}{\left| V \right| }\sum _{j\in V}q^T\cdot tanh(Wz_{j\cdot }^{\Phi _i}+b). \end{aligned}$$

Furthermore, a softmax function is used to normalize $w^{\Phi _i}$ as a semantic attention weight. Suppose the semantic attention weight for meta-path $\Phi _i$ is $\beta ^{\Phi _i}$, then

$$\begin{aligned} \beta ^{\Phi _i}=\frac{exp(w^{\Phi _i})}{\sum ^{3}_{j=1}exp(w^{\Phi _j})}, \end{aligned}$$

which represents the contribution of the semantic based on meta-path $\Phi _i$ to the classification task. Obviously, the higher $\beta ^{\Phi _i}$ is, the more important its semantic information is. For different tasks, $\beta ^{\Phi _i}$ may be different.

Finally, the weight $\beta ^{\Phi _i}$ in the attention network is used as a coefficient to integrate embedding vectors $Z^{\Phi _i}$, $i=1,2,3$ as a final embedding vector *Z*,

$$\begin{aligned} Z=\sum ^{3}_{i=1}\beta ^{\Phi _i}\cdot Z^{\Phi _i}. \end{aligned}$$

Obviously, vector *Z* has the same dimension as $Z^{\Phi _1}$, $Z^{\Phi _2}$ and $Z^{\Phi _3}$. It is the output vector of an HCAN layer.

### The model loss function

The final embedding vector *Z* of the last HCAN layer will go through a dropout layer to drop part of the features. Then, the feature embeddings after dropout are fed into an MLP with a softmax function to output a class vector $y'$, which is the prediction class value vector of the samples. Suppose *T* is a set of selected nodes, ${\left| T \right| }$ is the number of nodes in *T*, and *Y* is the set of classes. For node *l*, we use $y_i^l$ and ${y'}_i^l$ to represent its true class value and predicted value, respectively. We use the cross-entropy loss function to calculate the loss between the predicted value and the true value. Let $L_T$ be the loss of node set *T*, then it is calculated as follows

$$\begin{aligned} L_T=-\frac{1}{\left| T \right| }\sum _{l\in T}\sum _{Y}y_i^lln{y'}_i^l. \end{aligned}$$

## Results and discussion

In this section, the proposed method is tested on the ABIDE I dataset. FC features and non-image phenotype features of the selected subjects are used to construct a heterogeneous population graph.

For each sample node, 800 features selected from the 6105 functional connectivity features with the deep feature selection method (see [28]) are utilized as the node feature vector. The model is implemented in PyTorch. Training of the model uses a computer that contains an Intel (R) Core (TM) i5-9300 H CPU with 4 cores running at 4.00 GHz and 8 GB RAM, and an NVIDA GeForce GTX 1650MQ GPU with 896 CUDA cores and 4 GB GDDR5. During the model training, GPU acceleration and the early stop technique are utilized.

The parameters of the model are set as follows. The HCAN model includes two HCAN layers and an MLP. For each HCAN layer, the hidden size is 20, while in the MLP, the number of output units is 2. The Adam algorithm is used to optimize the model loss, where the learning rate is set to 0.005, and the weight decay is set to $5\times 10^{-4}$. For the dropout layer, the dropout rate is set to 0.6.

### Experiments on the ABIDE database

The proposed method is first tested on the whole dataset with 871 subjects. In the experiment, a 10-fold cross-validation schema that mixes data from all 17 sites while keeping the proportions between the different sites is used to evaluate the model performance. The average accuracy (ACC), sensitivity (SEN), specificity (SPE) and area under curve (AUC) are reported. The proposed HCAN method achieves an average ACC of 82.9%, SEN of 76.7%, SPE of 86.6% and AUC of 84.6%. The running time of performing 10-fold cross validation is 256 s.

Then, 5-fold cross-validation on each site is performed separately. The average ACC, SEN, SPE and AUC values are provided in Table 2. From the table, it can be seen that the SPE value of STANFORD is only 53.3% and the SEN value of SDSU is only 50%. The SEN values for both CALTECH and STANFORD are equal to 100%. This indicates that all the ASD subjects in the testing sets for the two sites were identified correctly. For CMU, it needs to be noted that there are only 11 subjects, and the ACC, SEN and SPE values are quite low (close to 60%). For all the datasets from different sites, the mean ACC, SEN, SPE and AUC values are 75.6%, 72.6%, 77.3% and 83.0%, respectively. In general, the proposed method performs well on the per site datasets.

**Table 2:** Average ACC, SEN, SPE and AUC values on individual site data using 5-fold cross-validation with our proposed method

Site	ASD/HC	Our proposed method			
		ACC (%)	SEN (%)	SPE (%)	AUC (%)
CALTECH	5/10	86.7	100.0	80.0	90.0
CMU	6/5	63.3	60.0	60.0	80.0
KKI	12/21	71.9	63.3	76.0	83.8
LEUVEN	26/30	83.7	81.0	85.5	81.9
MAXMUM	19/27	79.3	73.3	92.7	81.3
NYU	74/98	82.0	68.9	91.7	87.0
OHSU	12/13	72.3	63.3	80.0	86.7
OLIN	14/14	75.0	73.3	76.7	81.1
PITT	24/26	72.0	54.0	88.0	79.9
SBL	12/14	62.0	60.0	66.7	74.4
SDSU	8/19	74.0	50.0	85.0	82.5
STANFORD	12/13	75.7	100.00	53.3	76.7
TRINITY	19/25	73.3	80.0	68.0	78.0
UCLA	48/37	81.4	81.7	80.5	89.2
USM	43/24	75.1	83.1	60.0	78.4
UM	47/73	77.1	64.9	84.5	88.6
YALE	22/19	80.6	78.0	85.0	91.8
Mean		75.6	72.6	77.3	83.0

Column ‘ASD/HC’ shows the number of subjects with ASD and healthy controls, respectively

### Impact of model hyperparameters

This paper carries out experiments to study the impact of the model hyperparameters on the classification performance. In the HCAN model, the following three hyperparameters, namely, the number of HCAN layers, hidden size, and dropout rate, are investigated.

**Fig. 6:**
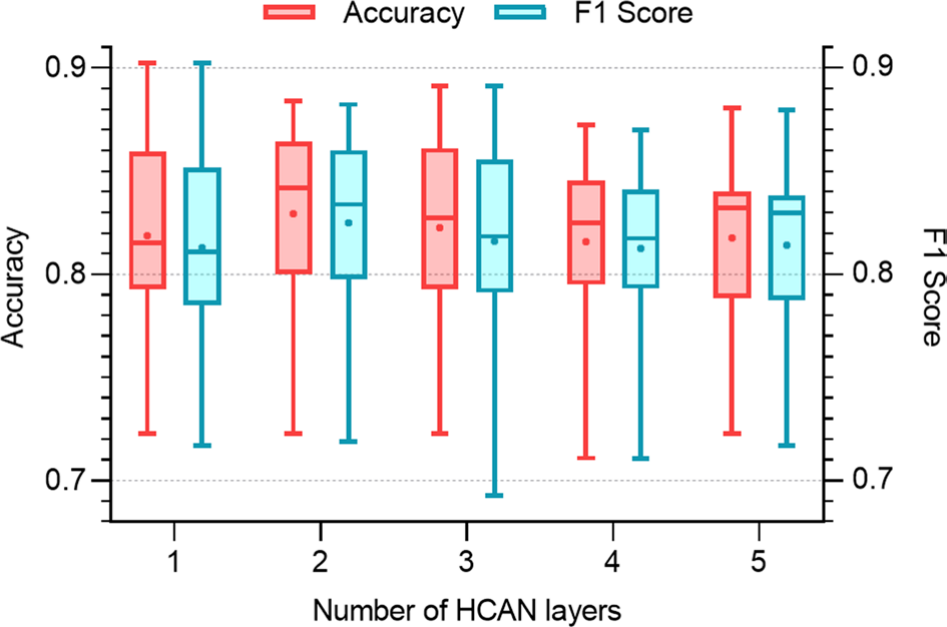
Impact of the HCAN layer number on the model performance

First, the relationship between the number of HCAN layers and the classification performance is explored. The number of HCAN layers is gradually increased from 1 to 5 while keeping the hidden size 20 and the dropout rate 0.6 unchanged. The accuracy and F1 score are computed. Figure 6 shows the comparative boxplot of accuracy and F1. For boxplots, the distribution of data based on a five-number summary including minimum, first quartile, median, third quartile, and maximum is displayed; also mean values in solid points are shown. When the number of HCAN layers increases from 1 to 2, the model performance improves significantly, while when the number of HCAN layers continues to increase, the model performance decreases.

**Fig. 7:**
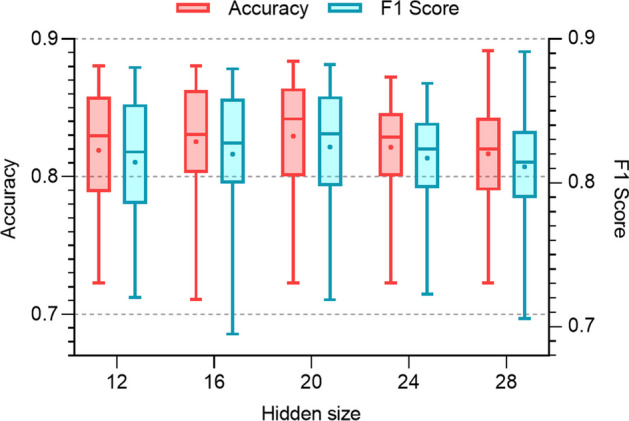
Impact of the hidden unit number on the model performance

Then, the impact of hidden size on the classification results is studied. The number of HCAN layers and the dropout rate are kept at 2 and 0.6, respectively. The hidden size is changed from 12 to 28 with a step size of 4. Figure 7 shows the impact of the hidden size. Before the hidden size increases to 20, the model performance is improved with increasing hidden size. However, once the hidden size is over 20, the model performance worsens.

In general, hyperparameters such as the number of layers and the hidden size in the network are related to the model complexity. A network with a larger number of layers or hidden size is of higher complexity. It seems that when the model complexity is low, increasing the model complexity can significantly improve the model performance, but when the model complexity reaches a certain degree, increasing the model complexity will cause overfitting and decrease the model performance.

**Fig. 8:**
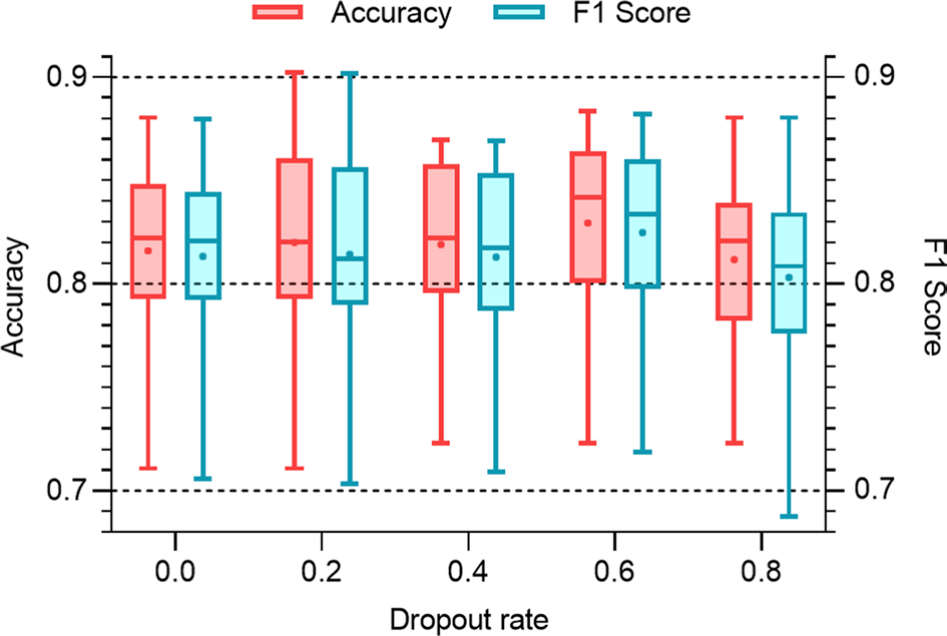
Impact of the dropout rate on the model performance

Finally, the influence of the dropout rate on the model performance is investigated. Dropout can be used to improve the model performance by reducing overfitting. The dropout rate is changed from 0 to 0.8 with a step size of 0.2, while the number of HCAN layers and hidden size are kept at 2 and 20, respectively. Figure 8 shows the change of accuracy and F1 score with the dropout rate. Both the accuracy and F1 score achieve the highest value when the dropout rate is equal to 0.6. However, when the dropout rate is over 0.6, the model performance decreases significantly due to the loss of feature information.

### Comparison with other methods

In our previous work [28], it was shown that the GCN method with deep feature selection is superior to some machine learning methods for the classification of ASD. In this work, the same comparisons are not repeated. Instead, to show the superior performance of our method, this paper compares the proposed method with some deep learning methods, i.e., MLP, HAN [30], GCN [28] and ASD-Diagnet [14].

In order to establish a fair comparison, all the above methods are implemented on the same computer and use the same 800 selected functional connection features. The same training and testing sets are used in the 10-fold cross-validation for all the methods. The parameters of MLP, HAN and GCN are optimally selected based on the grid-search method. In the MLP, 3 hidden layers, 16 hidden neurons and a dropout rate of 0.2 are set; In the GCN, 1 hidden layer and a dropout rate of 0.3 are set, and the graph weight matrix is constructed as described in [28]. In the HAN model, 2 HAN layers and 1 MLP layer are used; the output vector dimension for each HAN layer is 20; the output vector dimension of the MLP layer is 2; and the dropout rate is 0.6. For the MLP, HAN and HCAN models, a learning rate of 0.005 and weight decay of $5\times 10^{-4}$ in the Adam optimizer are used. For ASD-DiagNet, the code from https://github.com/pcdslab/ASD-DiagNet were downloaded, and the same parameters as the ones in [14] were used.

The average ACC, SEN, SPE and AUC values, as well as their standard deviations, are calculated. The running time for each method is also recorded. The results are listed in Table 3.

**Table 3:** Comparative results of different methods on the whole ABIDE dataset with 871 subjects

Model	ACC (%)	SEN (%)	SPE (%)	AUC	Time (s)
MLP	78.1 ± 4.7	77.2 ± 4.9	79.8 ± 4.8	83.1 ± 3.1	156
GCN	79.5 ± 3.3	**78.3** ± **3.5**	81.2 ± 3.6	**84.8** ± **2.7**	186
HAN	64.4 ± 2.9	38.7 ± 16.5	85.5 ± 12.4	64.4 ± 5.2	1556
HCAN	**82.1** ± **3.3**	76.7 ± 4.8	**86.6** ± **3.6**	84.6 ± 3.1	256
ASD-DiagNet	66.4 ± 3.9	55.60 ± 11.5	75.6 ± 8.9	73.0 ± 5.3	1924

The highest average values of ACC, SEN, AUC, and SPE are indicated in bold

From the table, it can be seen that the ACC, SEN and AUC of the HAN method are the lowest compared to the other methods, while the computation time of the HAN is the largest. Therefore, the performance of HAN is the worst. GCN and MLP perform better than ASD-DiagNet and HAN in terms of ACC, SEN, SPE, AUC and computational time. The proposed HCAN method achieves the best performance with an average accuracy of 82.9% and an average SEN of 86.6%. It is superior to the MLP, GCN, and HAN methods. It takes 256 s for HCAN to finish the 10-fold cross-validation, which is longer than MLP (156 s ) and GCN (186 s). This is because HCAN is more complicated than the MLP and GCN.

In the literature, except for Shao et al. [28], other researchers, i.e., Mostafa et al [10], Hu et al. [15], Liu et al.[16], Brahim and Farrugia[17], Yin et al. [18], Parisot et al. [22] and Rakhimberdina et al. [24], have also used the same 871 subjects (consisting of 403 patients with ASD and 468 healthy controls) in the ABIDE I dataset to classify ASD patients and normal controls. Therefore, this paper also compares the proposed method with these methods and summarizes the comparative results in Table 4. In the table, ‘Reference’, ‘Method’, ‘Number of ROIs’ (used for constructing features), and ‘Accuracy’ are listed.

**Table 4:** ASD classification on the ABIDE dataset with 871 subjects

Reference	Method	Number of ROIs	Accuracy (%)
Mostafa et al. [10]	LDA	264	77.7
Hu et al. [15]	FCNN	116	69.8
Liu et al. [16]	DFC+SVM	116	76.8
Brahim and Farrugia [17]	GFT+SVM	360	60.9
Yin et al. [18]	AE+DNN	264	79.2
Parisot et al. [22]	GCN	111	69.5
Rakhimberdina et al. [24]	GCN-based ensemble	111	73.1
Shao et al. [28]	DFS+GCN	111	79.5
Our proposed method	HCAN	111	82.9

From Table 4, it can be concluded that the proposed method performs the best among all the above methods. To the best of our knowledge, this result is so far the best in the literature for ASD classification with the selected 871 subjects.

The experimental results show that integrating non-imaging data has an important influence on the classification performance of ASD. By using all potential phenotypic measures and introducing an attention mechanism, new aggregated important features can be extracted from the HCAN network; thus, the classification performance can be improved. It needs to be noted that since the GCN involved in the model can only be applied to data with graphs of a fixed structure, if new subjects need to be predicted, it is necessary to reconstruct the graph using the phenotypic information of all the subjects. This will result in a high computational cost, which is the main limitation of the proposed method.

## Conclusions

In this paper, a deep learning model, namely, the heterogeneous graph convolutional attention network model, is constructed. The model is based on a heterogeneous graph and integrates a GCN and an attention mechanism. It uses rs-fMRI data and phenotypic data to classify ASD. The model can effectively extract features from a heterogeneous graph by integrating semantic information of different meta-paths with an attention mechanism. Experimental results have shown that the proposed model outperforms other methods. It reaches the current state of the art.

## Data Availability

The datasets analysed during the current study are available from a world-wide multi-site database Autism Brain Imaging Data Exchange (ABIDE I) (http://preprocessed-connectomes-project.org/).
